# Analysis of the genotyping of circulating strains and characteristics of Glycoprotein E of Varicella-Zoster Virus in Shanxi Province, China

**DOI:** 10.1371/journal.pone.0355368

**Published:** 2026-08-03

**Authors:** Hongye Liu, Peiyao Lv, Runmeng Ma, Shuping Guo, Ping Zhang, Jiane Guo, Ruihong Gao, Jitao Wang

**Affiliations:** 1 Shanxi Medical University, Taiyuan, China; 2 Department of Dermatology, The First Hospital of Shanxi Medical University, Taiyuan, China; 3 Taiyuan Center for Disease Control and Prevention, Xiaodian District, Taiyuan, China; Universidade Federal da Bahia, BRAZIL

## Abstract

**Background:**

Varicella-zoster virus (VZV), a neurotropic and epidermotropic herpesvirus, causes varicella (chickenpox) upon primary infection and reactivates later in life to cause herpes zoster (HZ), which is associated with significant morbidity, particularly postherpetic neuralgia (PHN). VZV genotyping and characterization of key viral proteins, such as glycoprotein E (gE), are critical for understanding viral circulation, distinguishing vaccine from wild-type strains, and optimizing vaccine strategies. In China, clade 2 is the predominant VZV genotype, but data from Shanxi Province remain limited.

**Methods:**

Forty herpes fluid specimens from clinically diagnosed herpes zoster (HZ) patients in Shanxi Province were collected. Quantitative polymerase chain reaction (qPCR) was used to detect viral DNA for definitive diagnosis. Six single-nucleotide polymorphisms (SNPs) in the open reading frame (ORF) 22 and ORF 38 fragments of positive specimens were analyzed by PCR and Sanger sequencing to determine viral genotypes. Four SNPs in ORF 38 and ORF 62 were analyzed to distinguish between vaccine and wild-type strains. The full-length nucleotide sequences of the gE gene in VZV-positive clinical specimens were determined. Sequence analysis was performed using Sequencher 5.4.6 and MEGA 11.0.11 software, with comparative reference to VZV strains retrieved from GenBank, to analyze the genetic characteristics of the VZV gE in Shanxi Province. The potential impact of amino acid substitutions caused by nucleotide mutations on the function of the gE protein was predicted using PROVEAN. Mutations in the gE protein were visualized using AlphaFold2 and PyMOL 2.6.0.

**Results:**

All 40 specimens tested positive for VZV and were identified as wild-type clade 2 strains. Strain SX-17 exhibited a T → C mutation at position 107252. Notably, two strains (SX-01 and SX-04) lacked the 69424 SNP locus in ORF 38. gE gene sequencing revealed amino acid substitutions compared to the Dumas reference strain, including T40I (39 strains), H98P (SX-18), V489L (SX-04 and SX-05), and N529S (SX-08). PROVEAN analysis classified all mutations as neutral (scores > −2.5), with no impact on critical gE functional domains.

**Conclusions:**

The dominant VZV genotype in Shanxi Province is wild-type clade 2. A T → C mutation at position 107252 exists in clade 2 strains. The mutation sites of gE identified so far have not exerted a significant impact on the VZV functions, suggesting that VZV vaccination can serve as a critical measure for herpes zoster prevention in Shanxi Province.

## 1. Introduction

Varicella-zoster virus (VZV, subfamily: Alphaherpesvirinae, genus: Varicellovirus, species: Human alphaherpesvirus 3) is an epidermotropic and neurotropic virus with a highly specific human host [1]. It has a double-stranded DNA genome of approximately 125 kb and is the causative agent of varicella (chickenpox) and herpes zoster (HZ, shingles) [2]. The global burden of HZ is substantial, with an annual incidence of 5–10 cases per 1000 individuals aged ≥50 years. Notably, approximately 30% of the population will develop shingles during their lifetime [3]. While VZV reactivation is usually restricted to a single dermatome, approximately 10% − 18% of adults with HZ develop postherpetic neuralgia (PHN), a neuropathic pain syndrome [4]. After the HZ rash subsides, severe pain may persist for months or even years [5], significantly affecting the patient’s quality of life and ability to perform daily activities.

Standardizing nomenclature facilitates the global exchange and comparison of VZV genotyping data. The genotype classification of VZV proposed by Jensen in 2017 is widely adopted and includes clades 1–6, a provisional clade VIII, and a newly identified clade 9 [6]. The distribution of VZV genotypes varies significantly across regions [7,8]. For example, clades 1 and 3 are predominant in Europe, the United States, and New Zealand, while clade 2 dominates in East Asia, clade 5 in Africa, and clade 4 in Southeast Asia [7–9]. In China, clade 2 is the predominant genotype of VZV [10–14]; however, in recent years, other genotypes have emerged in various provinces [15–21]. To date, molecular epidemiological data from Shanxi Province remain scarce.

The gE of VZV, encoded by ORF 68 (1872 bp, 623 amino acids), is the most abundantly expressed glycoprotein in VZV. It plays a major role in viral replication and assembly, and also mediates cell-to-cell transmission of the virus and immune evasion [22]. VZV gE possesses strong immunogenicity. It contains B-cell and CD4 + T-cell epitopes [23], while the cytoplasmic tail (CT) interacts with host factors via motifs like AYRV (for trans-Golgi localization) and YAGL (for endocytosis) [24,25]. Given its ability to stimulate both humoral and cellular immune responses, VZV gE represents the most commonly chosen antigen for HZ vaccines. Currently, there are two approved and marketed vaccines for the prevention of HZ, which differ in terms of vaccine type, administration route, protective efficacy, and immunogenicity. ZOSTAVAXÒ (manufactured by Merck & Co., Inc., Kenilworth, NJ, USA) is a live attenuated VZV vaccine based on the Oka/Merck strain. This vaccine has an efficacy of 64% in preventing herpes zoster among people aged 60–69. However, its efficacy gradually declines with increasing age, dropping to 18% in individuals aged 80 and above [26–28]. Shingrix, a herpes zoster subunit vaccine with gE as its main component, has an efficacy exceeding 97% in reducing the risk of herpes zoster in people aged 50 and above [29–32]. Shingrix and ZOSTAVAXÒ have been introduced in Shanxi Province, serving as an important choice for the elderly to reduce the risk of HZ.

This study aims to characterize VZV genotypes in HZ patients from Shanxi Province using molecular epidemiological methods, discriminate between vaccine and wild-type strains to assess vaccine strain prevalence, further analyze gE sequence variations and their functional implications to provide a scientific basis for the application of VZV vaccine in Shanxi Province.

## 2. Methods

### 2.1. Specimen collection

Specimens were collected from the vesicle fluid of inpatients clinically diagnosed with HZ in the Department of Dermatology at the First Hospital of Shanxi Medical University from October 2023 to June 2024. This study was conducted in accordance with the guidelines of the Declaration of Helsinki, and the experimental procedures were approved by the Institutional Review Board and Human Research Ethics Committee of the First Hospital of Shanxi Medical University (approval number: KYLL-2023–222, approved on 5 June 2023). The study did not involve human participants or human experimentation; the only human material used was herpes fluid specimens collected from herpes zoster (HZ) patients for public health purposes. All inpatients provided written informed consent prior to their inclusion in the study. The individual in this manuscript has given written informed consent (as outlined in the PLOS consent form) to publish these case details. All specimens were stored at −80 °C after collection.

### 2.2. Viral nucleic acid extraction

VZV DNA was extracted from the specimens using the CDC-approved Viral DNA/RNA Extraction Kit (Xi’an Tianlong Science and Technology Co., Ltd.) following the manufacturer’s instructions. After automated extraction, the eluate was transferred to sterile nuclease-free microcentrifuge tubes. The purified DNA was either used immediately or archived at −80 °C for long-term preservation.

### 2.3. Detection of VZV DNA

The VZV Nucleic Acid Detection Kit (Beijing BioRegional Biotechnology Co., Ltd.) was employed to detect VZV nucleic acids. Each 20 µL premixed reaction system, prepared according to the manufacturer’s instructions, had 5 µL of specimen DNA added to it. Detection was performed on a CFX96 Real-Time PCR system (Bio-Rad, Hercules, California, USA), with the thermal cycling parameters set as specified in the kit’s instructions. Specimens with a Ct value ≤ 35 and a characteristic S-shaped amplification curve were deemed positive.

### 2.4. VZV genetic analysis and vaccine versus wild-type strain discrimination

The ORF 22, ORF 38, and ORF62 gene fragments were specifically amplified using the Takara Ex Taq™ kit (RR001A). Primers for ORF 22, ORF 38 and ORF 62 were obtained from previous studies [33,34]. Each PCR reaction mixture contained 5 µl of 10 × Ex-Taq buffer, 4 µl of 2.5 mM Ex-dNTP mix, and 0.25 µl of Ex-Taq polymerase, in addition to 20 pmol of each primer and 5 µl of sample DNA to make up to 50 µl with sterile nuclease-free water. The PCR reaction conditions were as follows: initial denaturation at 95 °C for 2 min; 30 cycles of 94 °C for 40 s, 55 °C for 40 s, and 72 °C for 1.5 min; and a final extension at 72 °C for 5 min. Bidirectional Sanger sequencing of PCR products was performed by Berry Genomics Co., Ltd. The sequencing results were assembled using Sequencher 5.4.6 (Gene Codes, Ann Arbor, MI). VZV genotyping was determined by alignment against the VZV Dumas reference strain (GenBank: X04370) through six signature SNP loci (37,902, 38,019, 38,055, 38,081, 38,177, 69,424). The discrimination between vaccine and wild-type strains was based on four previously reported SNPs, three of which were located within ORF 62 at positions 106262, 107252, 108111, and the fourth one at 69349 in ORF 38 [35].

### 2.5. Analysis of gE

#### 2.5.1. gE characterization.

Three overlapping fragments spanning ORF 68 were amplified under modified cycling parameters: 95 °C for 2 min; 30 cycles of 95 °C for 1 min, 50 °C for 1 min, 72 °C for 45 s; 72 °C for 10 min final extension. The sequences of the PCR primers were referenced from a previous study [36]. Sequences were compared against the reference strain (X04370) from GenBank using BioEdit 7.2 for multiple sequence alignment and MEGA 11.0.11 for phylogenetic reconstruction.

#### 2.5.2. Prediction of mutation effect calculation.

The PROVEAN (Protein Variation Effect Analyzer) online tool was employed to predict the potential impact of amino acid substitutions caused by nucleotide mutations on protein function. Mutation information was submitted to the PROVEAN online server (http://provean.jcvi.org/), which integrates a large number of homologous sequences from the NCBI protein database. By comparing the mutant sequence with the homologous sequences, a prediction score was generated. A threshold criterion of ≤ −2.5 was used: mutations with a prediction score of ≤ −2.5 were classified as “deleterious,” indicating a significant potential impact on protein function; whereas mutations with a score > −2.5 were determined to be “neutral,” suggesting a relatively minor impact on protein function.

#### 2.5.3. Visualization of gE Mutation sites.

The three-dimensional (3D) structure model of the gE protein was predicted using the AlphaFold2 online platform (https://alphafoldserver.com/). The obtained model was then imported into the molecular visualization software PyMOL for 3D visualization and labeling of mutation sites in the gE protein.

## 3. Results

### 3.1. Basic information of patients

A total of 40 hospitalized HZ patients whose disease course was within 3 days were enrolled from October 2023 to June 2024. All patients had no history of herpes zoster vaccination. Among them, there were 16 male and 24 female patients, with a mean age of 61.44 years. Nerve involvement included the trigeminal nerve in 14 cases (including 1 case of Ramsay-Hunt syndrome), the intercostal nerve in 11 cases, the lumbosacral nerve in 9 cases, and the unilateral limb nerve in 6 cases, with all patients presenting typical clinical manifestations of HZ and varying degrees of neuralgias. Comorbidities comprised hypertension (13 cases), diabetes mellitus (15 cases), rheumatoid arthritis (2 cases), Sjögren’s syndrome (1 case), renal insufficiency (4 cases), and tumors (3 cases: 1 lymphoma, 1 prostate cancer, 1 lung cancer). Treatment protocols followed the Chinese Expert Consensus on Herpes Zoster Diagnosis and Treatment (2022 Edition), including antiviral therapy (intravenous acyclovir or oral brivudine) and analgesic therapy (gabapentin, pregabalin, or topical lidocaine patches). A three-month follow-up after discharge showed postherpetic neuralgia (PHN) in 6 cases (15%), and one patient with lumbosacral nerve involvement developed an eczematous isomorphic reaction in the original herpes area two weeks after herpes resolution. Clinical characteristics are summarized in Table 1.

**Table 1:** Clinical characteristics of HZ patients.

Item	Specific classification		Value
Gender	Male		16 cases
	Female		24 cases
Age	Minimum		32 years old
	Maximum		85 years old
	Average		61.44 years old
Nerve Involvement Site	Trigeminal Nerve		14 cases (including 1 case of Ramsay – Hunt Syndrome)
	Intercostal Nerve		11 cases
	Lumbosacral Nerve		9 cases
	Unilateral Limb Nerve		6 cases
Vaccination History	None		40 cases
Underlying Disease	Hypertension		13 cases
	Diabetes Mellitus		15 cases
	Rheumatoid Arthritis		2 cases
	Sjögren’s syndrome		1 case
	renal insufficiency		4 cases
	Tumor	lymphoma	1 case
		prostate cancer	1 case
		lung cancer	1 case
Complication	Post - Herpetic Neuralgia (PHN)		6 cases
	Eczematous Isomorphic Reaction (in the original herpes area, in the patient with lumbosacral nerve involvement)		1 case

### 3.2. Results of qPCR detection

The DNA extracted from 40 suspected herpes zoster specimens was subjected to qPCR, and all specimens tested positive.

### 3.3. Genotyping

The sequencing results of the PCR products from ORF 22 and ORF 38, compared with the reference strains of different types, showed that all 40 specimens belonged to clade 2. The sequences of specimens SX-01 and SX-04 did not show the 69424 locus. See Table 2.

**Table 2:** Nucleotide contents at polymorphic positions within ORF 22 and ORF 38 of VZV genomes obtained from clinical specimens of 40 patients.

VZV strain	Clade	ORF 22 SNP sites					ORF 38 SNP site
		37902	38019	38055	38081	38177	69424
Dumas/MSP	1	A	G	T	A	G	G
POka/vOka	2	G	G	C	C	A	G
HJ0/03–500	3	A	G	T	A	G	A
M2DR/8	4	A	A	C	C	A	G
CA123	5	A	G	T	C	G	G
Var160	6	A	G	T	C	A	A
1483/2005	Ⅷ	A	G	T	C	A	G
457/2008	9	A	G	C	A	G	A
SX-01	2	G	G	C	C	A	/
SX-04	2	G	G	C	C	A	/
The other 38 specimens excluding SX – 01 and SX – 04	2	G	G	C	C	A	G

Note: All genome positions are based on the published sequence for the VZV reference strain: Dumas (GenRank accession number X04370); “/”means not detected

### 3.4. Identification of vaccine strains and wild-type strains

The analysis results of four SNP sites (69349, 106262, 107252, and 108111) in ORF 38 and ORF 62 indicated that all 40 VZV strains were wild-type strains. The nucleotide contents at the four polymorphic positions were consistent with those found in Dumas and YC01 strains among the clinical isolates, consisting of A-T-T-T, except for isolate SX-17, which presented with the variant A-T-C-T. See Table 3.

**Table 3:** Nucleotide contents at polymorphic positions within ORF 38 and ORF 62 of VZV genomes obtained from clinical specimens of 40 patients from Shanxi province.

VZV strain	Wild-type Strain/Vaccine Strain	ORF 38 SNP Site	ORF 62 SNP Sites		
		69349 (*Pst*Ⅰ)	106262 (*Sma*Ⅰ)	107252 (*Nae*Ⅰ)	108111 (*Bsr*Ⅰ)
vOka	Vaccine Strain	G	C	C	C
Baike	Vaccine Strain	G	C	C	C
VarilRix	Vaccine Strain	G	C	C	C
VariVax	Vaccine Strain	G	C	C	C
MAV06	Vaccine Strain	G	C	C	C
Dumas	Wild-type Strain	A	T	T	T
YC01	Wild-type Strain	A	T	T	T
pOka	Wild-type Strain	G	T	T	T
SX-17	Wild-type Strain	A	T	C	T
The other 39 specimens excluding SX – 17	Wild-type Strain	A	T	T	T

### 3.5. gE protein analysis

The gE protein sequences of VZV in 40 specimens were obtained, with the corresponding NCBI sequence numbers ranging from PV268493 to PV268532. A comparative analysis was conducted on the 40 VZV specimens tested in this study, as well as the domestic (VOKA-BK) and imported (Varilrix-1) live attenuated varicella vaccine strains. It was found that, except for SX-10, all other specimens had a base mutation of C119T at the 115926 site, which led to the mutation of the 40th amino acid from threonine to isoleucine (T40I). This mutation also occurred in all reported vaccine strains and wild-type strains of other genotypes except Clade 1. Additional mutations included H98P (SX-18), V429E (SX-04), R489P (SX-04 and SX-05), and N529S (SX-08). The PROVEAN analysis results showed that the prediction scores for all evaluated mutations were greater than −2.5, indicating that all mutations in the gE protein were predicted to be neutral. The mutation sites are shown in Table 4. None of the identified mutations were located within the critical regions of the gE CT (amino acids 568–571;582–585;593;595;596;598). The 3D structural visualization of the gE protein and the annotated mutation sites are presented in S1 Fig.

**Table 4:** Analysis of gE sequences and PROVEAN analysis of mutations in 40 VZV strains.

VZV strain	Position	Base	gE mutation			PROVEAN score	Prediction (cutoff = −2.5)
	(Dumas)		Change (nucleotide/amino acid)	Type of amino acid change			
SX-01 ~ SX-09; SX-11 ~ SX-40;vOka-BK; VarilRiX-1	115926	C	C119T;T40I a	neutral p→non-p	neutral→hb	0.240	Neutral
SX-18	116100	A	A293C;H98P b	basic→non-p	hb→neutral	−0.042	Neutral
SX-04;SX-05	117272	G	G1465C;V489L b	non-p→non-p	neutral→neutral	0.675	Neutral
SX-08	117393	A	A1586G;N529S b	neutral p→neutral p	hb→hb	0.342	Neutral
SX-07	115814	A	A7C;T3P b	neutral p→non-p	hb→neutral	−0.292	Neutral

Note: ^a^ Mutations reported previously [8,37,38].

^b^Mutations unique to Shanxi Province.

1. All genome positions are based on the published sequence for the VZV reference strain:Dumas (GenBank accession number X04370)

2. Only changes relative to Dumas are indicated

3. The type of amino acid changes is indicated in terms of polarity (p: polar) and hydrophobicity (hl:hydrophilic; hb: hydrophobic; N/A: not applicable)

## 4. Discussion

As a common viral skin disease in dermatology, HZ primarily affects the elderly population [3,39]. Its predilection sites are the intercostal nerves (53%), cervical nerves (20%), trigeminal nerves (15%), and lumbosacral nerves (11%) [40]. The HZ patients enrolled in this study had a mean age of 61.44 years, with the trigeminal nerve being the most frequently involved site (14 cases), which may be associated with the higher proportion of severe or complex cases collected from inpatient departments. VZV DNA was detected in all 40 enrolled patients via PCR, indicating that HZ can be definitively diagnosed based on typical clinical manifestations. However, in atypical cases, such as zoster sine herpete or neuralgia, the preceding rash, detection of virus DNA (e.g., PCR) should be conducted when feasible to exclude other potential etiologies. Additionally, the absence of prior herpes zoster vaccination in all patients suggests the current status of low vaccination coverage for HZ in Shanxi Province.

In China, the predominant genotype of VZV is clade 2 [10–13]. In Xinjiang and Tibet, molecular epidemiological studies of VZV have found that, in addition to the clade 2 genotype, clade 1, clade 3, and clade 5 genotypes are also prevalent among varicella and herpes zoster cases [15,18]. In Guangdong, in addition to the clade 2 genotype, clade 4 and clade 5 genotypes have also been identified [18,21]. In Beijing, the presence of clade 1, clade 3, and clade 4 genotypes has been reported. Clade 5 has been sporadically observed in Shenzhen [16] and Ningbo [20]. In Changchun, VZV strains with characteristics of both clade 1 and clade 3 have been detected [19], likely due to viral recombination or mixed infections. These variations may be attributed to geographical distribution, population genetic background, or viral evolution. The genotypes of 40 VZV strains from Shanxi Province were classified into clade 2, consistent with previous reports in other provinces. Notably, two strains (SX-01 and SX-04) lacked the 69424 SNP locus in ORF 38, a rare feature that has not been reported in clade 2 before. The functional significance of this deletion remains unclear. It may represent a novel subclade or sequencing artifact, warranting validation in larger sample cohorts.

Differentiating vOka vaccine strains from wild-type VZV is vital for viral surveillance. In previous studies, genotyping assays for distinguishing vOka vaccine strains from wild-type VZV mainly relied on a single SNP locus [41–48]. However, a number of studies have shown that vOka vaccine preparations contain a mixture of genetically distinct viral haplotypes, which renders single-locus identification unreliable for strain discrimination. Accordingly, four SNPs within ORF38 (position 69349) and ORF62 (positions 105705, 106262, and 107252) are recommended for reliable VZV strain identification [34]. SNP analysis of ORF38 and ORF62 in our study classified all 40 isolates as wild-type strains, with no vaccine strains identified. Notably, strain SX-17 in our study bore the identical T → C substitution at 107252. This strain was isolated from a 75-year-old elderly patient with impaired immunity, who only presented typical clinical manifestations of herpes zoster. Previous studies have reported two wild-type VZV strains harboring the 107252 T → C mutation, one belonging to Clade 3 [34] and the other to Clade 5 [49]. The present study first identifies this mutation within Clade 2. This mutation causes a serine-to-glycine substitution in IE62, the major transactivator encoded by ORF62, which may alter viral neuroinvasiveness and latent infection capacity. It remains unclear whether this mutation is a transient variant inside the host or a stable adaptive mutation formed during human-to-human transmission of the virus; larger sample sizes and long-term continuous surveillance are needed.

VZV gE is composed of four main domains: a signal peptide (SP) at the N-terminal end, an ectodomain, a transmembrane domain (TM), and finally, a CT at the C-terminal end [50]. Fowler and Garcia-Valcarcel’s research identified that the regions eliciting the strongest cellular immune responses are amino acid positions 11–30, 71–90, 91–110, and 106–125 [51,52]. Liu Jian et al. demonstrated that amino acid residues 121–135 of the gE protein constitute a key antigenic epitope for neutralizing antibodies [53]. Based on these findings, amino acid mutations within these regions may lead to disruption or alteration of T-cell epitopes, enhancing the virus’s potential for immune escape and causing a decline in vaccine protective efficacy. It is noteworthy that the SX-18 in this study exhibited an H98P amino acid mutation. According to the Kyte-Doolittle hydrophobicity scale [54] and the amino acid isoelectric point table, it was found that after mutation, the positive charge at this site decreased and the hydrophobicity increased, directly altering the local charge distribution and hydrophobicity. In this study, 39 out of the 40 tested VZV specimens (excluding SX-10), along with the domestic (vOka-BK) and imported (VarilRiX-1) varicella live-attenuated vaccines, all harbored a C115926T base mutation. This mutation resulted in a threonine-to-isoleucine substitution at amino acid position 40 (T40I). This identical mutation has been documented in previously reported vaccine strains, suggesting that it lacks genotypic specificity and virulence correlation. The VZV gE antigenic epitopes involved in this study thus remain highly conserved.

The CT of gE contains three motifs crucial for its subcellular localization. The AYRV sequence (amino acids 568−571) guides gE to the TGN, while the YAGL sequence (amino acids 582−585), similar to YXXL endocytosis motifs, facilitates gE internalization. Additionally, an “acid patch” consisting of two serines (S593 and S595) and two threonines (T596 and T598) participates in gE trafficking [24]. Raquel et al.’s study found that modifications to the CT of gE significantly impact its subcellular localization, and this impact is closely correlated with the immunogenicity of the VZV mRNA vaccine. It is inferred that mutations at the above-mentioned sites in the CT of gE may affect the virus’s latency-reactivation balance and immune escape capability. The sequence analysis of the gE glycoprotein showed that multiple amino acid substitutions were identified in all 40 wild-type strains, including T40I, H98P, V489L, and N529S. These mutations altered amino acid polarity or hydrophobicity, but all were predicted as functionally neutral by PROVEAN (scores > −2.5), indicating no significant impact on protein structure or function. Furthermore, none of these mutations affected the key functional domains of the CT region of the gE protein.

Considering the low vaccination coverage and the presence of neutral gE mutations, it is recommended that universal vaccination be implemented for adults ≥ 50 years to reduce the incidence of HZ and the risk of PHN in Shanxi province. While increasing the vaccination rate, it is also essential to continue monitoring the circulating strains of VZV. This will allow us to promptly understand the transmission dynamics of VZV in the local area, providing references for the prevention and control of VZV and the development and optimization of novel vaccines.

While providing the first molecular data from Shanxi, this study has limitations: a single-center sample (40 cases), a lack of vaccination coverage data, and uncharacterized functional effects of the ORF 38 deletion. Future research should include multi-center sampling, correlate genotypes with clinical outcomes, and investigate gE-specific immunity in vaccinated populations.

## 5. Conclusions

The dominant genotype of VZV in Shanxi Province is clade 2, and the prevalent strains are wild-type. This study first identified the 107252 T → C mutation in wild-type Clade 2 strains, and whether it causes a decrease in pathogenicity requires further expanded sampling and long-term continuous surveillance. To date, no mutations affecting the structure and function of gE proteins have been detected. This suggests that VZV vaccination can serve as a critical measure for preventing HZ in Shanxi Province.

## Supporting information

S1 Fig The 3D structural visualization of the gE protein and the annotated mutation sites. (PDF)
