# Supplementary material for: Analysis of the genotyping of circulating strains and characteristics of Glycoprotein E of Varicella-Zoster Virus in Shanxi Province, China
Source: PLoS One. 2026 Aug 3;21(8):e0355368. doi: 10.1371/journal.pone.0355368 (PMC13432107; doi:10.1371/journal.pone.0355368)

Figure A. The 3D structural visualization of gE protein and the annotated mutation sites

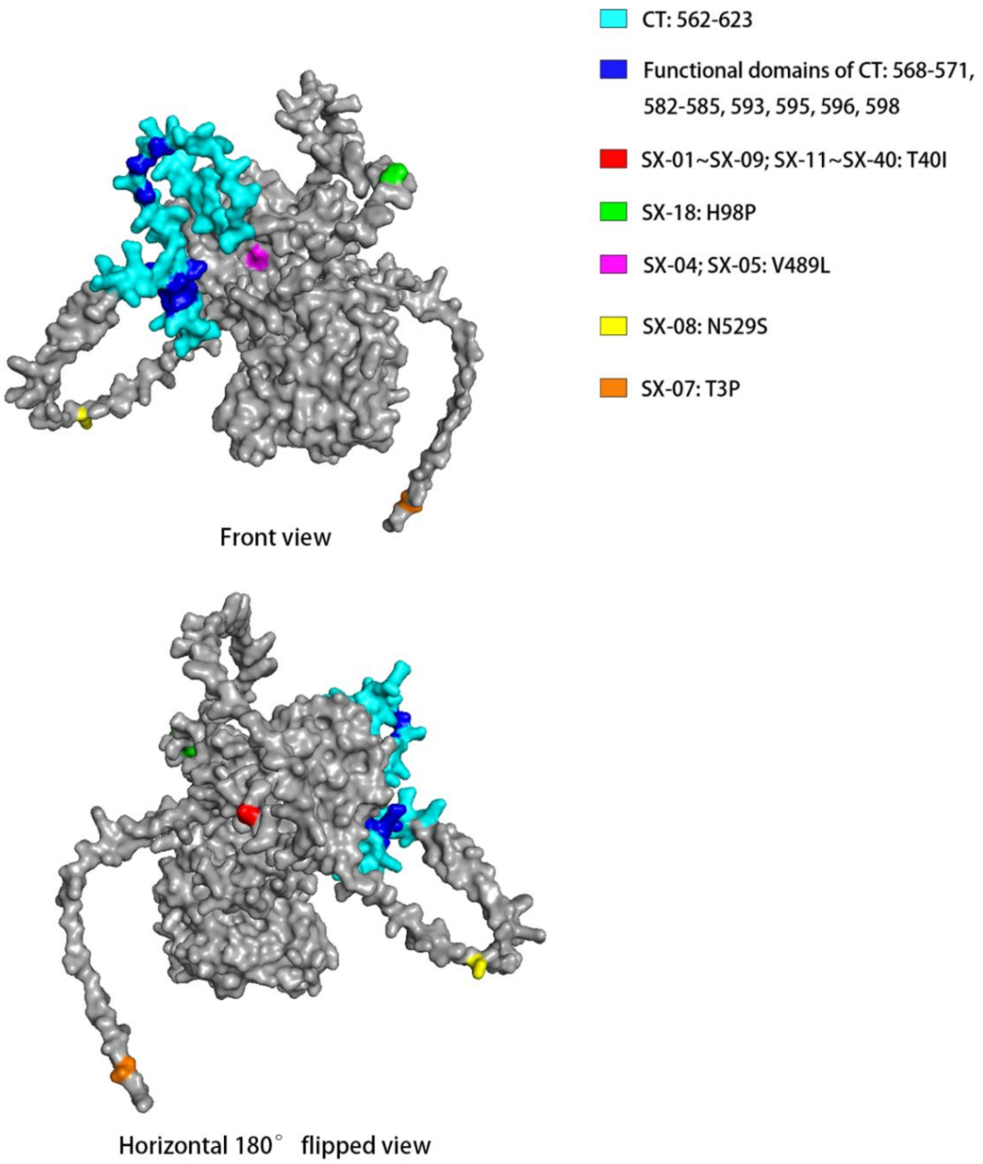

Figure B. 3D structures of gE proteins with different mutations and their mutation sites (in red)

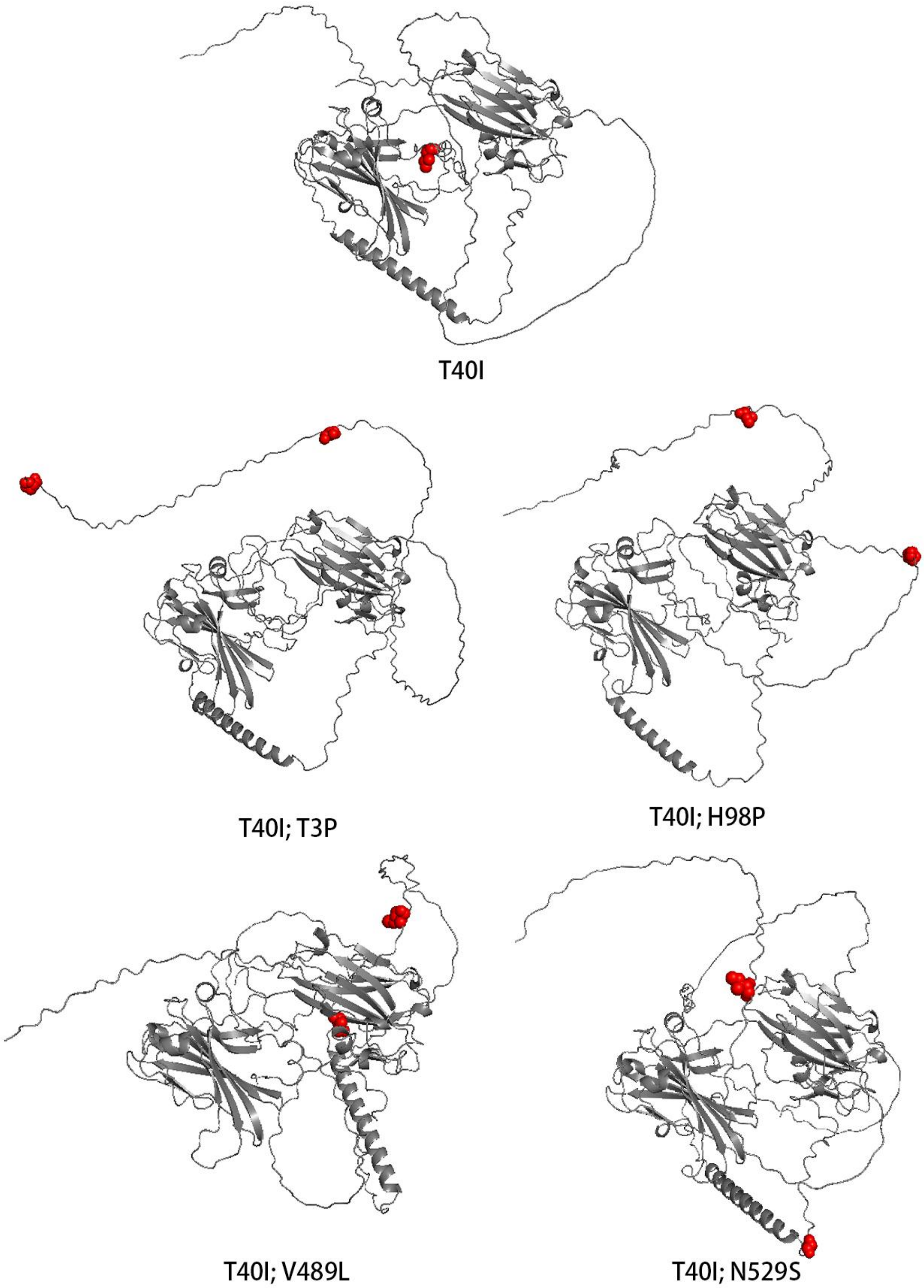

Supplement: S1 Fig — (PDF) [file pone.0355368.s001.pdf]
